# Supplementary material for: Maximal glycolytic flux modulates metabolic thresholds independent of maximal oxygen uptake
Source: Eur J Appl Physiol. 2026 Jun 6;126(9):4903–18. doi: 10.1007/s00421-026-06270-1 (PMC13541831; doi:10.1007/s00421-026-06270-1)
Supplement: Supplementary file 1 [file 421_2026_6270_MOESM24_ESM.docx]

**Maximal Glycolytic Flux Modulates Metabolic Thresholds Independent of Maximal Oxygen Uptake**

Benedikt Meixner^1,2,3^ ([benedikt.meixner@fau.de](mailto:benedikt.meixner@fau.de) ), Peter Leo ^4^ ([leo@ridewithpassion.tirol](mailto:leo@ridewithpassion.tirol) ) & Billy Sperlich^1^ ([billy.sperlich@uni-wuerzburg.de](mailto:billy.sperlich@uni-wuerzburg.de))

Correspondence to:

Benedikt Meixner

Orcid: 0000-0001-7044-9426

Integrative and Experimental Exercise Science & Training,

Department of Sport Science, Julius-Maximilians-Universität Würzburg, Judenbühlweg 11, 97082 Würzburg, Germany

[benedikt.meixner@fau.de](mailto:benedikt.meixner@fau.de)

**Running Title:** Glycolytic Flux Modulates Thresholds

**Abbreviations**

| P_2_ | Power output at 2 mmol·L^-1^ lactate concentration |
| --- | --- |
| P_4_ | Power output at 4 mmol·L^-1^ |
| P_+0.5_ | Power at minimum value + 0.5 mmol·L^-1^ lactate concentration |
| P_+1.5_ | Power output at minimum value + 1.5 mmol·L^-1^ lactate concentration lactate concentration |
| BM | Body mass |
| Fat_max_ | Power output associated with maximal fat oxidation |
| FFM | Fat-free mass |
| P_VT1_ | Power output at VT_1_ |
| P_VT2_ | Power output at VT_2_ |
| P_LaWmin_ | Power output at minimal lactate equivalent |
| LT | Lactate treshold |
| MAD | Mean absolute deviation |
| MLSS | Maximal lactate steady state |
| PFO | Peak fat oxidation rate |
| *v*La_max_ | maximal lactate accumulation rate |
| VT_1_ | Ventilatory threshold 1 |
| VT_2_ | Ventilatory threshold 2 |
| ΔLa | maximal capillary blood lactate accumulation |
| Rpm | Revolutions per minute |

**Abstract**

Purpose: This study examined how maximal glycolytic flux, indexed by the peak lactate accumulation rate (*v*La_peak_) from a 15-s sprint, relates to lactate- and gas-exchange derived endurance thresholds at a given V̇O_2_max in trained cyclists.

Methods: Fifty cyclists (30 men, 20 women) completed two laboratory visits: a ramp test with verification to determine V̇O_2_max, and on a separate day, a 15-s all-out sprint with repeated post-exercise capillary blood sampling to determine peak lactate accumulation (ΔLa) and derived *v*La_peak_, followed by a 3-min step-increment test with lactate and gas-exchange measurements.

Results: Partial correlations controlling for V̇O_2_max revealed moderate-to-strong negative associations between *v*La_peak_ and selected lactate- and gas-exchange–derived thresholds (r = –0.37 to –0.59, p < 0.01), whereas absolute and relative 15-s work showed consistently weaker or no relationships (r = 0.06 to –0.48). No association was observed between *v*La_peak_ and peak fat oxidation. In general linear models, adding *v*La_peak_ to models with V̇O_2_max and sex as predictors significantly increased the explained variance in threshold power (ΔR^2^ ≈ 0.06 to 0.20). Sex contributed additional but consistently smaller effects.

Conclusion: These findings indicate that the power output at several metabolic thresholds originates from the interaction of maximal oxidative and glycolytic flux. As such, *v*La_peak_ seems to be a promising parameter that complements traditional measures like V̇O_2_max and helps to improve metabolic profiling and exercise prescription. However, longitudinal studies are needed to verify these findings.

**Introduction**

Maximal oxygen uptake (V̇O_2_max) reflects the highest rate to produce adenosine triphosphate (ATP) through oxidative phosphorylation and is widely recognized as a primary determinant of endurance performance (Lucía et al. 1998; Joyner 1991; Hargreaves and Spriet 2020). Mechanistically, V̇O_2_max  emerges from the integrated limits of pulmonary diffusion capacity, cardiac output, blood oxygen-carrying capacity, and skeletal muscle oxidative capacity (Bassett and Howley 2000).

However, for many endurance sports, maximal lactate steady state (MLSS) and associated lactate (LT) or ventilatory thresholds (VT) are often more critical for performance differentiation than V̇O_2_max per se (Lucía et al. 1998; Joyner 1991; Hagberg and Coyle 1983; Farrell et al. 1979; Coyle et al. 1991; Coyle et al. 1988; Yoshida et al. 1987; Faude et al. 2009). Over the last decade, the mechanistic interpretation of lactate and gas‐exchange thresholds has shifted away from the original “anaerobic” or dysoxic view (Poole et al. 2021). It has been argued that the rise in blood lactate concentration and excess carbon dioxide (CO_2_) at the so‐called anaerobic threshold is better explained by an increased rate of glycolysis and lactate release into the blood relative to lactate removal, rather than by widespread muscle anoxia (Poole et al. 2021).  In this view, thresholds emerge when glycolytic flux and the associated proton load exceed the rate of oxidative and buffering processes to stabilize blood lactate and acid–base status during progressively increasing exercise (Brooks 2018; Brooks 2010; Ferguson et al. 2018).

This flux-based perspective highlights glycolysis as a key determinant of threshold behaviour. A plausible explanation is that glycolytic flux at submaximal intensities is not constant across individuals, but scales with each person’s maximal glycolytic capacity – analogous to expressing oxygen uptake at threshold as a fraction of V̇O_2_max (Mader 2003; Mader and Heck 1986). Consequently, athletes with a higher maximal glycolytic flux may reach a given threshold at a lower external power output because, at the same submaximal workload, their glycolytic system is recruited more readily and provides a greater fration of total ATP

The metabolic model proposed by Mader (Mader 1984; Mader and Heck 1986) provides a mechanistic-mathematical model for the interaction between (maximal function of) glycolytic and oxidative pathways (Brooks et al. 2022a; Ferguson et al. 2018) to explain the genesis of tresholds (Heck and Wackerhage 2024). It describes lactate steady-state equilibrium based on the interplay between two primary rates: the maximal rate of oxidative phosphorylation (V̇O_2_max) and maximal glycolytic flux (*v*La_max_). In practice, conceptual *v*La_max_ is estimated from the empirical maximal lactate accumulation rate during a short, maximal sprint (termed *v*La_peak_) and is regarded as a proxy for maximal glycolytic activity in the absence of direct cellular measurements (Wackerhage et al. 2022; Wackerhage et al. 2025; Heck et al. 2003). Within this framework, higher *v*La_max_ is predicted to shift the lactate–power curve leftward at a given V̇O_2_max, lowering the power sustainable at lactate-defined thresholds and altering substrate-use patterns (Wackerhage et al. 2022; Wackerhage et al. 2025; Beneke 2003b).

These model predictions have important implications for metabolic flexibility (San-Millan and Brooks 2018) and for the prevention and treatment of non-communicable diseases (Esteves and Stanford 2024), yet the connection between maximal glycolytic flux and thresholds determined during incremental exercise tests has so far only been addressed in simulations, not in experimental human data. In particular, the model predicts that a lower maximal glycolytic flux reduces pyruvate availability at lower intensities, thereby promoting greater fat oxidation as long as oxidative lactate clearance exceeds lactate production at a given workload (Beneke 2003b, a; Brooks et al. 2022b; Sablain et al. 2025).

Consequently, as stated above, a lower maximal glycolytic flux should theoretically be associated with higher peak fat oxidation (PFO) and with a rightward shift of the workload eliciting maximal fat oxidation (Fat_max_) (Jeukendrup and Achten 2001; Beneke 2003b, a; Mader 2003), but this has not been demonstrated empirically using direct experimental estimates of maximal glycolytic flux.

One practical and increasingly used method to estimate maximal glycolytic flux in vivo is based on a short-duration all-out sprint test. In this case, a 10–30 s sprint (Heck et al. 2003) is combined with repeated post-exercise blood lactate sampling to determine the maximal peak lactate accumulation (ΔLa) and the corresponding peak accumulation rate (*v*La_peak_, mmol·L^-1^·s^-1^), which can be interpreted as an empirical marker of maximal whole-body glycolytic flux (Margaria et al. 1964; di Prampero and Ferretti 1999; Ferretti 2023, 2015).

In contrast to the modelling approach of metabolism, incremental step tests provide an accessible method for identifying metabolic thresholds and substrate oxidation patterns in many endurance sports, thereby informing training interventions and performance diagnostics (Faude et al. 2009; Jamnick et al. 2020). Yet, the theoretical link between *v*La_peak_ and thresholds derived from such tests, and its consequences for substrate-use profiles, remains largely untested. Recent work even reported that V̇O_2_max was the strongest predictor of a fixed lactate threshold, whereas *v*La_peak_ showed little independent explanatory value (Fischer et al. 2025), raising questions about how maximal glycolytic flux and oxidative capacity jointly shape endurance exercise metabolism. *v*La_max_ and the related empirically determined index *v*La_peak_ have been consistently linked to sprint performance, with strong correlations reported between maximal glycolytic flux and short-duration power output (Clark and Macdermid 2025; Held et al. 2023; Meixner et al. 2025b; Meixner et al. 2025c; Meixner et al. 2024b). Based on Mader’s model, however, markers of maximal glycolytic flux should also help explain endurance-related variables, potentially providing more physiologically meaningful information than mechanical metrics alone despite their close interdependence (Meixner et al. 2024b).

Moreover, several studies have highlighted potential sex differences in metabolic profiles, including distinct patterns of fat oxidation, lactate thresholds relative to peak power, and glycolytic capacity between men and women (Esbjörnsson-Liljedahl et al. 2002; Benitez-Muñoz et al. 2024; Benítez-Muñoz et al. 2025; Thron et al. 2024; Meixner et al. 2024b; Quittmann et al. 2022). These disparities likely reflect differences in hormonal milieu, muscle fiber composition, and enzyme activities that influence substrate utilization and glycolytic flux. Accordingly, sex may modify the relationship between maximal glycolytic flux, thresholds, and substrate use, with implications for performance diagnostics and training prescription (Oosthuyse and Bosch 2006; Devries 2016; Boisseau and Isacco 2022; Tarnopolsky and Ruby 2001).

Given that V̇O_2_max on its own is a strong independent predictor of metabolic flexibility (San-Millan and Brooks 2018) and exercise metabolism (Burtscher et al. 2023; Millet et al. 2023) in general, it may mask relationships with glycolytic parameters, it is essential to account for its influence in statistical analyses. Therefore, this study aims to: (i) evaluate correlations between sprint-derived glycolytic flux parameters (i.e. *v*La_peak_, as an empirical marker for maximal glycolytic flux) and various established lactate (P_2_; P_+0.5_; P_4_; P_+1.5_; P_LaWmin_), ventilatory (P_VT1_; P_VT2_) thresholds and fat oxidation (Fat_max_) ; (ii) quantify the explanatory power of a 15-s sprint test and therefrom derived *v*La_peak_ for these metabolic thresholds when added to models that already include V̇O_2_max ; and (iii) examine whether these relationships differ between men and women.

We tested the hypothesis that the implications of maximal glycolytic flux extend beyond short-duration exercise, i.e. that maximal glycolytic flux, indexed by *v*La_peak_ from a 15-s sprint, would be negatively associated with first and second thresholds, independent of V̇O_2_max, and would provide greater explanatory value than purely mechanical measures of sprint performance. We further hypothesized that these associations would be present in both sexes and across a selection of metabolic thresholds, but that their magnitude might differ between men and women.

**Methods and materials**

Participants

A cohort of fifty (*n* = 30 male, *n* = 20 female) healthy and experienced cyclists with more than 3 years of cycling exercise (>2 sessions per week) was recruited for this study. All participants were experienced in road cycling with clipless pedals and cycled regularly as exercise. Prior to the study, the participants were informed of the protocol and gave their written informed consent to participate. All procedures were approved by the ethical committee of Exercise Science & Training of the Faculty of Human Sciences (EV2024/1–1004) and conducted in accordance with the Declaration of Helsinki (Harriss and Atkinson 2009). Participant characteristics are given as Mean±SD in Table 1.

*****insert table 1 around here*****

Study Design

Two experimental visits (T1 and T2) to the laboratory were required, which were at least 48 h apart and completed within a 7-day period. Participants underwent a sprint test as familiarization and a ramp protocol during the first visit; a sprint test and a step incremental test during the second visit. All testing was performed in the laboratory of the department of sport science and sport in Erlangen from April to December of 2022. The overall study design is illustrated in Figure 1.

An a priori target of N=50 participants (≥1/3 female) was set based on feasibility constraints (McCrum et al. 2022). In addition, for the planned multiple linear regression analyses adjusting for V̇O_2_max and sex, assuming α=0.05 (two-sided), N=50 provides approximately 80% power to detect an incremental effect of *v*La_peak_ of *f^2^* ≈ 0.18 when added to the covariate model (Abt et al. 2025; Lakens 2022).

*****insert figure 1 around here*****

All participants were instructed to keep a nutrition diary and to repeat their usual diet for each visit within the 24 h before each experimental visit (Jeacocke and Burke 2010). In addition, all were instructed to stay adequately hydrated, to eat a carbohydrate-rich meal (i.e., a banana and a jam sandwich) no less than 3 h before each visit, and to refrain from caffeine consumption on the day of testing. Each participant received 35 g of a carbohydrate mixture (IsoFast, DextroEnergy, Krefeld, Germany) dissolved in 500 ml of water to drink *ad libitum* during warm-up and recovery periods. The main intent of this procedure was to ensure adequate hydration and carbohdydrate availability after the warmup, sprint and recovery period for the step test in line with recommendations (Thomas et al. 2016); based on previous work, minimal to no influence on thresholds is to be expected (Rotstein et al. 2007; Aandahl et al. 2021).

All cycling tests were conducted on a Cyclus2 ergometer (RBM, Leipzig, Germany) and their own personal road bike. The Cyclus2 is an electromagnetically braked ergometer and measures power with an accuracy error of 2% according to the manufacturer. Considering that all participants performed the tests in a similar gear, between-bike losses of mechanical power output induced by drivetrain friction are considered minimal (Dahmen et al. 2011; Lanaspeze et al. 2025). All cyclists used their own shoes and pedals for all tests. A warmup of 10min at a load of 1.5 W · kg^-1^ BM^-1^ was used for all visits to the laboratory.

Body composition

During the first visit, stature was measured as well as body mass (BM) and fat-free mass (FFM) of all participants, employing eight-electrode impedance analysis (InBody 720, Biospace, Des Moines, Iowa, USA)(McLester et al. 2020).

15s-sprint

The 15s all-out cycle sprint was performed in a seated position utilizing the large chainring (if applicable) of the participant's bike and the 15-tooth cog of the ergometer. A short, unloaded pedalling phase < 3 s and rpm < 30 was employed before the sprint, recording of the test started when cadence surpassed 30 rpm. The ergometer software was set to isokinetic mode and 130 rpm (Adam et al. 2015; Nitzsche et al. 2018). Capillary blood samples of the left earlobe were sampled twice during the resting period, after the warm-up and once directly after the sprint as well as every minute for 9 min after the 15-s cycle sprint. We ensured capillary blood lactate values reached peak values by continuing measurement if no clear peak was reached by the end of the 9^th^ minute. Resting lactate concentration was defined as the mean of the two pre-exercise samples. *v*La_peak_ was calculated as indicated in the formula (Heck et al. 2003; Wackerhage et al. 2025)

$$v\mathrm{La}_{peak}=\frac{La_{peakpost}-La_{pre}}{t_{test}}=\frac{\Delta La}{15s}$$

Ramp protocol

To determine V̇O_2_max and peak power output (PPO_ramp_), all participants performed a ramp protocol in T1. The participants began cycling at 100 W for 2 min with a freely chosen cadence, after which the load increased by 25 W every 30 s (Adam et al. 2015). The ramp ended when volitional exhaustion was reached, or the cadence dropped by more than 10 rpm. Following 3-min active recovery at 75 W, a verification phase at 100% of the load at the last fully completed 30 s until volitional exhaustion was performed (Midgley and Carroll 2009).

Incremental step protocol

To determine power output at various endurance thresholds, participants underwent a incremental step protocol (T2). The participants began the test with a freely chosen cadence but were advised to maintain their regular cadence. The test started at 100 W, with the load increasing by 25 W every 3 min. Three participants wished to start at 75 W based on previous results. The test concluded when volitional exhaustion was reached or when cadence consistently dropped by more than 10 rpm. Capillary blood samples of the left earlobe were sampled before the start of the step test and in the last 15 s of every step.

Gas Exchange and fat oxidation measurement

Participants were fitted with a Hans Rudolph V2 mask (Hans Rudolph, Inc, Shawnee, KS, USA), and expired gases along with breathing volume were analyzed using a Cosmed Quark CPET system (Cosmed Srl, Rome, Italy). Gas and volume analyzers were calibrated before each test using precision gas (16% O_2_ and 5% CO_2_) and a volume pump, following the manufacturer’s instructions (Airgas Therapeutics, Plumsteadville, PA, USA). Gas exchange parameters were averaged over 10-s intervals.

In line with the manufacturer’s recommendations, we employed a mixing chamber setup for the ramp test and breath-by-breath measurements for the step incremental test. V̇O_2_max was calculated as the highest moving average over 30 s in the ramp test as indicated in Omnia software (v2.4.2., Cosmed Srl, Rome, Italy)(Nolte et al. 2023; Iannetta et al. 2018), matching the step duration of ramp increments.

Only the final 30 s of each step were considered for analysis and fat oxidation rates were determined using standard nonprotein stoichiometric equations for moderate to high intensity according to Jeukendrup and Wallis (2005). VT_1_ and VT_2_  was determined following the method described by (Keir et al. 2022) and assessed by two independent experienced researchers in exercise testing and prescription who were blinded to the identity of the athletes and the aim of the study. Power output at VT_1_ and VT_2_ was linearly interpolated based on the test duration.

Lactate concentration measurement and threshold determination

Lactate concentrationwas measured amperometric-enzymatically employing Biosen C-Line (EKF Diagnostics, Barleben, Germany) from 20 μL earlobe capillary samples. Lactate values were interpolated using third-order polynomial regression analysis, with all regressions attaining adequate goodness of fit (*R*^2^ ≥ 0.97). Based on the regression, the following thresholds were calculated as power output: the minimal equivalent of lactate (P_LaWmin_), lactate concentration at the minimal lactate value during the step test +0.5 mmol·L^-1^ (P_+0.5_) respectively +1.5 mmol·L^-1^ (P_+1.5_) and thresholds at fixed lactate concentrations of 2 mmol·L^-1^and 4 mmol·L^-1^ (P_2_ resp. P_4_)(Pallarés et al. 2016; Hartmann et al. 1990).

Statistical analysis

All data were collected and exported in Microsoft Excel. Statistical analysis was performed employing jamovi (The jamovi project, v2.6.26.0) and Python 3.11.7, using the numpy 1.26.4, statsmodels 0.14.0 and matplotlib 3.8.0 packages. Normality of the lactate accumulation parameters, power outputs, and V̇O_2_max was assessed using the Shapiro–Wilk test, without requiring further transformation, except for power output at Fat_max_. Median and mean absolute deviation (MAD) are therefore given for Fat_max_ (Bouza 2013). The level of significance (*α*) was set to 0.05 for all statistical analyses.

Partial Pearson correlations were conducted to examine the relationship between parameters derived from the sprint test (primarily *v*La_peak)_ and endurance thresholds, while controlling for V̇O2max.

We fitted a model a General linear model (GLM) for all threshold through a base model of V̇O_2_max and sex. The categorical predictor sex was coded with 0 = females, 1 = males for inclusion in the model. We expanded this model by adding *v*La_peak,_ 15s-work and 15s-work/FFM in separate models. Incremental model contribution of each sprint-derived predictor beyond the base model was quantified as the change in explained variance (ΔR^2^) between nested models (e.g., M_0_ vs. M_1_) Statistical significance of ΔR^2^ was tested using partial F-tests comparing the full and reduced ordinary least squares models. As secondary analyses, we evaluated whether 15s-work or 15s-work/FFM provided additional explanatory value when added to the *v*La_peak_ model.

**Results**

Results of sprint test and selected endurance thresholds for males, females and the all participants are presented in table 2.

*****insert table 2 around here*****

*v*La_peak_ displayed significant negative correlations to all thresholds but not to PFO or Fat_max_ in the partial correlation analysis(figure 2).

*****insert figure 2 around here*****

Generalized linear models displayed a significant effect of V̇O_2_max and of *v*La_peak_. Estimate of *v*La_peak_ was negative. Sex had a significant effect for all lactate-derrived thresholds except for Fat_max_ and consistently had smaller effect sizes than *v*La_peak_. For P_VT1_ and P_VT2_, we display the influence of *v*La_peak_ without consideration of sex in figure 3. Models containing *v*La_peak_ displayed consistently higher ΔR^2^ than those integrating 15s-work or 15s-work/FFM (table 3). Nested model comparisons adding either 15s-work or 15s-work/FFM to M_1_ with *v*La_peak_ yielded small but significant improvements for 15s-work/FFM and thresholds P_LaWmin_, P_+0.5_, P_+1.5_ and P_4_ (table 4). Figures of other thresholds as well as full model parameters are supplied in supplementary material.

*****insert table 3 around here*****

*****insert figure 3 around here*****

*****insert table 4 around here*****

**Discussion**

The primary aim of this study was to characterize how maximal glycolytic flux a sprint-derived via a 15-s sprint (*v*La_peak_) relates to several endurance thresholds at a given V̇O_2_max, and thereby to describe the interplay between glycolytic and oxidative pathways in vivo. The key findings were:

i) *v*La_peak_ showed stronger negative partial correlations with selected lactate- and gas-exchange–derived endurance thresholds than absolute or relative 15-s power, after statistically accounting for V̇O_2_max;
ii) including *v*La_peak_ alongside V̇O_2_max in linear models substantially increased the variance explained in threshold power, consistent with the notion that the lactate–power relationship is shaped by the combined limits of maximal oxidative and maximal glycolytic flux rather than by V̇O_2_max alone;
iii) sex was identified as a significant independent factor for most lactate-derived thresholds, although its contribution was consistently smaller than that of *v*La_peak_, suggesting that sex-related differences in glycolytic flux, substrate use, and lactate kinetics modulate the expression of thresholds but do not replace the primary role of maximal flux parameters.

Maximal glycolytic flux and endurance thresholds

The results of our study indicate that there is a significant negative association between the sprint-derived peak lactate accumulation rate (*v*La_peak_), as a marker of maximal glycolytic flux, and selected endurance thresholds. This supports the concept outlined in Mader’s model (Mader 2003; Mader 1984; Mader and Heck 1986) that these thresholds emerge from the interplay between maximal oxidative and maximal glycolytic flux rather than from V̇O_2_max alone and warrants further elucidation of the mechanisms behind this relationship. The comparable associations for P_VT1_ , P_VT2_ and Fat_max_ suggest the *v*La_peak_ - threshold link is not specific to lactate curve fitting, but also manifests in gas-exchange–based breakpoint behavior.

In the primary modelling framework, adding *v*La_peak_ to the base model containing V̇O_2_max and sex (M_0_ → M_1_) consistently improved model fit across all threshold outcomes, with ΔR^2^ ≈ 0.06 – 0.20 (partial F-tests: all p<0.01). This indicates that *v*La_peak_ captures a meaningful portion of inter-individual variability in threshold power that is not explained by V̇O_2_max alone, supporting the view that glycolytic flux capacity contributes to threshold behaviour beyond differences in maximal aerobic power.

In secondary nested comparisons, we tested whether sprint work metrics provide additional explanatory value once *v*La_peak_ is already included. Adding 15-s work to the *v*La_peak_ model produced negligible changes in explained variance (ΔR^2^ ≈ 0.00 – 0.006) and was not significant across outcomes (partial F-tests: p>0.05), suggesting that absolute sprint work does not contribute materially beyond *v*La_peak_ in this dataset. By contrast, adding 15s-work/FFM yielded small but occasionally significant improvements for selected lactate-derived thresholds (ΔR^2^ ≈ 0.012–0.025, partial F-tests: p<0.05 in several outcomes), indicating a modest additional component associated with size-normalized sprint work that is not fully captured by *v*La_peak_. Overall, these results reinforce *v*La_peak_ as the dominant sprint-derived predictor within the framework, while suggesting limited incremental information from work/FFM for some thresholds.

Although previous studies have shown that work performed during a 15-s all-out sprint is closely associated with body composition and lactate accumulation (Meixner et al. 2025b; Meixner et al. 2025c; Meixner et al. 2024b), *v*La_peak_ demonstrates stronger and more consistent correlations with endurance-related markers than either absolute or relative 15-second sprint work. Therefore, we conclude that the peak lactate accumulation rate during a 15-s sprint serves as a more specific indicator of maximal glycolytic flux and is more sensitive than power output during the sprint, which depends on other factors such as body composition, mechanical efficiency, or neuromuscular activation (Bundle and Weyand 2012). This is especially noteworthy as glycolytic contribution and work accomplished during a 15-s sprint are strongly linked (Meixner et al. 2024b) and is positively related to 60 s power output (Clark and Macdermid 2025). Previously, this negative relationship between a marker of maximal glycolysis and endurance thresholds had been shown only in theory. Additionally, lactate measurement for the determination of glycolytic flux is essential, as only capillary blood lactate concentrations provide a meaningful representation of glycolytic activity across all active musculature beyond what power output in the 15s-sprint alone can indicate, despite the close linkage of the two parameters and the energetic significance of lactate accumulation (Meixner et al. 2024b; Ferretti and di Prampero 2026).

Previous studies have highlighted the utility of all-out sprint tests and the associated maximal capillary blood lactate accumulation as integrative markers of endurance performance and metabolic profile (Ji et al. 2021; Quittmann et al. 2022; Hauser et al. 2014; Poffé et al. 2024). As theorized in Mader’s equations (Mader and Heck 1986), the maximal lactate accumulation rate in active muscles plays a crucial role in determining the fractional utilization of V̇O2max. The present data extend these theoretical and modelling observations – in a simplified way and through statistical methods to alleviate methodological challenges – by showing that a directly measured *v*La_peak_ relates to multiple lactate- and gas-exchange–derived thresholds after accounting for V̇O_2_max, thereby experimentally supporting the idea that submaximal glycolytic behaviour at thresholds is partly determined by an individual’s maximal glycolytic flux.

Peak Fat Oxidation

No significant correlation was found for peak fat oxidation (PFO). PFO plays an important role in longer duration endurance exercise where the athlete is not metabolically but energetically limited. However, this finding in our data, which is not supported by the Mader model, may be caused by several factors: i) the determination of PFO is not a very reliable testing procedure (Chrzanowski-Smith et al. 2020); ii) the previously performed sprint test with considerable accumulation of blood lactate may influence the capacity for fat oxidation (San-Millan and Brooks 2018) even though lactate levels were lowered before the start of the incremental test; iii) other factors such as time of day, habitual nutrition and muscle glycogen levels as well as age may influence the measurement of PFO more than V̇O_2_max and *v*La_peak_ (Amaro-Gahete et al. 2018; Maunder et al. 2018; Purdom et al. 2018)(Amaro-Gahete et al. 2018; Maunder et al. 2018; Purdom et al. 2018). Thus, the absence of a significant association between maximal glycolytic flux and PFO in the present study should be interpreted cautiously and may reflect methodological noise and acute test sequencing rather than a true absence of interaction between glycolytic and oxidative pathways at lower intensities.

Statistical instead of physiological modeling

We tested a sprint-derived marker of maximal glycolytic flux (*v*La_peak_) as an informative descriptor of the oxidative–glycolytic interplay at endurance thresholds, rather than to fully implement the original physiological modelling approach of Mader (Mader 2003; Mader 1984). We rely on statistical modelling instead of full physiological modelling as described by Mader (Mader 2003; Mader and Heck 1986) to show that *v*La_peak_ is more strongly related to threshold power than power-based sprint parameters, supporting its value as a glycolytic flux marker. Several reasons were responsible for this approach. The *v*La_max_ parameter is theorized in the level of muscle metabolism and a necessity for the model simulation, but cannot readily be measured directly and is instead assumed to be approximated by *v*La_peak_ (Wackerhage et al. 2025).

Further, the determination of an alactic time component remains unresolved. As power-based parameters have been shown to exhibit only poor to moderate reliability (Meixner et al. 2024a), a fixed alactic time frame could in principle resolve this issue (Wackerhage et al. 2025). However, there is still debate about this approach and about which fixed value to use, especially in relation to total test duration (Meixner et al. 2024a; Langley et al. 2025; Langley et al. 2024; Dunst et al. 2023). Nonetheless, an assumption of this time frame (even if set to 0 s) is needed to attain a model-based rate of maximal lactate accumulation in addition to total test time and ΔLa.

Further methodological issues persist in the estimation of maximal glycolytic rate as a proxy measure for maximal glycolytic activity: it remains unclear whether the test should be optimized for maximal lactate accumulation or for maximal mechanical performance (Dunst et al. 2025; Haase et al. 2024). The optimal duration for the test is also unresolved, as some authors consider durations of 10 to 12 s better suited because higher vLa_peak_ values are attained (Langley et al. 2024; Porter and Langley 2025(Quittmann 2025)). We consider not the highest possible blood lactate values to be optimal, but those with the highest informational value across metabolic demands

While conceptual markers of muscle glycolytic activity (*v*La_max_) are required for simulation and insertion into the model, experimental studies can only access peak parameters of lactate accumulation at the whole-body level through concentration measures in capillary blood (Wackerhage et al. 2025). Since its inception, it has been suggested that the actually measured parameter is only an estimation (Heck et al. 2003). Nonetheless, our *v*La_peak_, calculated as ΔLa divided by sprint duration, follows to the recently proposed calculation method (Wackerhage et al. 2025) and therefore represents an empirical marker of maximal net glycolytic flux rather than a fully modelled *v*La_max_ of the active muscle.

It is also important to recognize that capillary blood lactate accumulation following the sprint test may be influenced by cardiovascular and hemodynamic function. The transport of lactate from muscle to blood is mediated by monocarboxylate transporters and changes in their expression may alter the appearance of lactate in capillary blood (Dubouchaud et al. 2000; Thomas et al. 2005). Additionally, systemic lactate uptake and clearance by tissues such as the heart, brain, liver, and skeletal muscle may contribute to variations in capillary blood lactate accumulation and are themselves influenced by endurance training status (Brooks 2009; Dubouchaud et al. 2000; Leija et al. 2025). Therefore, our measurements of maximal glycolytic flux reflect net accumulation rather than true production of lactate, and a direct measure of intramuscular lactate production rate is not possible through capillary blood measurements alone. Furthermore, lactate clearance capacity might be a confounding factor for both endurance thresholds and post-sprint lactate accumulation. It also needs to be mentioned that lactate accumulation after an all-out sprint test does not represent a peak instantaneous value of glycolysis, but merely the mean value over the duration of the test.

The original approach by Margaria (Margaria et al. 1964) to determine maximal lactate accumulation rate used a series of supramaximal exercise tests. Interestingly enough, this rate was similar across participants and uphill running grades and comparatively close the the average value of our male participants. The onset of lactate accumulation depended on Phosphocreatine (PCr) stores, which is something we could not replicate in our own study in the sprint test under creatine supplementation (Meixner et al. 2025c). Nonetheless, it may be that the sprint test is sensitive enough to accurately display a maximal rate of glycolysis in a specific exercise mode irrespective of PCr stores to achieve predictive value for endurance exercise. Therefore, we refrained from the calculation of a fully parameterized *v*La_max_ for insertion into simulation models and opted for a simplified statistical modelling approach, using *v*La_peak_ as an empirical marker of maximal glycolytic flux and examining its relationship with thresholds and fat oxidation at a given V̇O_2_max. In this sense, the present work should be viewed as an experimental constraint and augmentation of physiological modelling, rather than as a replacement for it. In this context, our results complement recent predictive modelling work in cycling (e.g., Wahl & Ji’s MuDo-PD) by providing an empirical constraint on whether a sprint-derived glycolytic marker adds explanatory information beyond V̇O_2max and threshold anchors (Wahl and Ji 2026). In line with the discussion and potential influence of alactice time on vlamax (Dunst et al. 2023) and hence simulation results, we chose to forgoe this discussion and eliminate this parameter by setting it equal to zero. For physiological modelling, alactic time remains a primary calibration problem while this is not a problem in regression analysis.

Due to these methodological issues, the Mader model and the parameter *v*La*_max_* so far have failed to gain broader international scientific recognition and application, preventing wider understanding and exploration of the mechanisms described in the model. The results of our study show that a simple empirically derived glycolytic flux parameter *v*La_peak_ already provides substantial information for constructing a metabolic profile of an athlete. Furthermore, it provides experimental evidence that metabolic behaviour under incremental loads reflects the interplay between maximal oxidative and maximal glycolytic rates. We stress that our findings, obtained through statistical modelling, should be interpreted cautiously as qualitative support for flux-based models of thresholds and as a starting point for the refinement and wider application of physiological models of exercise metabolism.

Sex differences

The results of our study indicate that sex is an additional factor, independent of *v*La_peak_ and V̇O_2_max, in explaining variation in endurance thresholds. In the general linear models, men showed higher threshold power than women at a given V̇O_2_max and *v*La_peak_, although the effect size of sex was consistently smaller than that of *v*La_peak_. Previous studies have described differences in maximal lactate accumulation between males and females (Meixner et al. 2024b; Quittmann 2025). It remains unclear whether this is predominantly influenced by muscle architecture (Esbjörnsson-Liljedahl et al. 2002), training and neuromuscular activation (Bundle and Weyand 2012), or sex-related differences in lactate distribution and elimination, together with differences in fat-free mass (FFM) between the sexes (Tripp et al. 2025).

It has also been reported that females have a higher fractional utilization of thresholds in relation to their V̇O_2_max (Benítez-Muñoz et al. 2024; Benitez-Muñoz et al. 2024; Benítez-Muñoz et al. 2025). This could mechanistically be explained by their muscle fiber distribution (Esbjörnsson-Liljedahl et al. 2002) and their lower maximal lactate accumulation (Mader and Heck 1986; Meixner et al. 2024b). However, in our data, regression results display the opposite pattern, i.e. at the same V̇O_2_max and *v*La_peak_, threshold power was slightly higher in men. The study by Benítez-Muñoz et al. (2025) employed only a step incremental test, whereas in our study, V̇O_2_max was obtained from a ramp test and thresholds were determined in a separate incremental step test (Meixner et al. 2026). The same befalls other studies in this area that employ only one test for both threshold and maximal variables (Johansen et al. 2025; Meixner et al. 2026), but interestingly enough, these studies may be further benefit from including *v*La_peak_ as additional and mechanistical information. Therefore, methodological differences, including test protocols and definitions of thresholds, are potentially the source of the disparity observed in our study. In addition, our models did not explicitly adjust for mechanical efficiency or muscle mass, which may mediate part of the sex effect on threshold power.

Unlike Fischer et al. (2025), we did not assess efficiency because of the comparatively short duration of 3 min per step. We stress that we determined predictors in separate specific tests, unlike their approach of deconstruction thresholds through a single step incremental test. It is likely that efficiency could enhance statistical modelling and should also be included as a factor in physiological modelling (Sablain et al. 2025), but from our perspective, should be evaluated in an independend diagnostic procedure. Future work that combines measurements of maximal glycolytic flux, mechanical efficiency and muscle mass that consider biological sex differences may therefore better resolve how sex modulates the relationship between oxidative and glycolytic fluxes at endurance thresholds. Our data suggests that the effect of interaction of rates is larger than differences rooted in sex of individuals, but this factor may modulate the behavior of this interaction.

Physiological interpretation

At the cellular level, maximal glycolytic flux is likely governed by the combined activity of rate-limiting glycolytic enzymes, particularly phosphofructokinase (PFK) and other key control steps in glycolysis (Zuo et al. 2021; Boscá and Corredor 1984; Mor et al. 2011) , together with the capacity of monocarboxylate transporters (MCT1, MCT4) to export lactate from producer fibres and import it into oxidative tissues (Kitaoka et al. 2012; Bonen 2001; Seyedi et al. 2024). These processes determine how rapidly pyruvate and cytosolic NADH are generated and how much of this reducing-equivalent load can be handled by mitochondrial oxidation versus lactate formation and export (Spriet et al. 2000; Vigh-Larsen et al. 2021). In this context, our finding that higher sprint-derived maximal glycolytic flux (*v*La_peak_) is associated with lower threshold power at a given V̇O_2_max may represent a systems-level manifestation of glycolytic–oxidative competition for pyruvate and NADH turnover, as envisaged by contemporary lactate shuttle and threshold concepts (Brooks 2009, 2018; Poole et al. 2021; Glancy et al. 2021). At higher maximal glycolytic flux, glycolytic activation during incremental exercise will more rapidly increase pyruvate and NADH supply, so that the point at which lactate appearance outpaces disappearance is reached at a lower external workload, consistent with a leftward shift of the lactate–power relationship.

Importantly, this *push* interpretation of glycolysis is only one plausible perspective. An alternative (and not mutually exclusive) view is that glycolytic flux is largely *pulled* by ATP demand and regulatory signals, i.e., by the mismatch between ATP demand and the combined capacity of oxidative phosphorylation and phosphagen buffering, with *v*La_peak_ reflecting glycolytic responsiveness rather than a primary upstream driver. This interpretation is consistent with classical control concepts in which glycolytic rate is modulated by metabolites linked to ATP turnover (e.g., ADP/P_i_) alongside constraints imposed by oxidative capacity. Thus, our data are compatible with multiple mechanistic framings while pointing to the same functional outcome – a leftward shift of the lactate–power relationship at higher *v*La_peak_.

Practical implications and Perspective

The results of our study indicate experimental support for the informational value of maximal glycolytic flux and its influence on endurance-related markers at a given V̇O_2_max. Despite the methodological challenges of assessing maximal glycolytic rate, the value of the model for practice does not only lie in the simulation of metabolism but rather in the qualitative understanding of the underlying flux-based mechanisms. When recognizing maximal glycolytic flux of the working musculature as an important influence on endurance performance, training interventions can specifically aim for a directional change in this parameter. A simple 15-s sprint with post-exercise lactate sampling, yielding *v*La_peak_, may therefore complement traditional V̇O_2_max and threshold testing when constructing an athlete’s metabolic profile.

Traditional lactate curve analysis fails to provide more mechanistic understanding behind threshold genesis through the metabolic profile of an athlete (Bleicher et al. 1998). In contrast, combining V̇O_2_max and *v*La_peak_ explicitly frames thresholds as emergent properties of the interaction between maximal oxidative and maximal glycolytic fluxes, offering a more mechanistic basis for interpreting individual differences and training responses. Future studies should focus on the interplay of *v*La_peak_ and V̇O_2_max through training interventions, quantifying how much of the change in endurance performance and thresholds is attributable to adaptations in oxidative versus glycolytic flux, and whether this balance should be targeted differently across sexes, sports and competitive demands (Quittmann 2025). Furthermore, it is unclear how *v*La_peak_ may develop in different training programs and if any change in *v*La_peak_ is reflected in longitudinal lactate curve analysis. Although our cohort consisted of trained cyclists, the principle that the interplay of maximal aerobic flux and maximal glycolytic flux modulate the energetic contributions across intensities may also be relevant for understanding exercise intolerance and metabolic inflexibility in clinical populations.

**Strengths and Limitations**

A key strength of this study is its sample size and inclusion of both male and female cyclists, allowing us to examine sex as an independent factor in the relationship between maximal glycolytic flux and endurance thresholds. Additionally, prior familiarization sessions enhanced the reliability of sprint test outcomes (Meixner et al. 2024a).

A wide array of endurance thresholds exists (Jamnick et al. 2020; Faude et al. 2009) and these provide information relevant to training zones, but not necessarily a direct approximation of maximal lactate steady state. Our study did not assess this equilibrium state, which also depends on definitions as well as measurement accuracy and reliability. Nonetheless, the Mader model originally calculates steady-state conditions based on lactate production and removal.

Cadence is a potential confounding factor in the determination of efficiency (Marsh et al. 2000) with additional effects on endurance thresholds, especially with regard to Mader’s model (Dunst et al. 2025). This was not controlled for in our analysis, as self-selected cadence most likely reflects the participants’ habitual pedalling patterns best. Furthermore, *v*La_peak_ was derived from capillary blood lactate and reflects net accumulation rather than true intramuscular production flux (as discussed above), and the test order (sprint preceding the step test) as well as pre-exercise carbohydrate intake may have influenced substrate use and fat oxidation. We did not directly assess muscle enzyme activities, MCT expression or mitochondrial density, so the proposed mechanistic links between *v*La_peak_, enzyme or transporter function and threshold behaviour remain inferential. Finally, our cohort consisted of trained cyclists, which limits generalizability of the present findings to other sports and less-trained or clinical populations but represents a suitable model population for proof-of-concept.

**Conclusion**

*v*La_peak_ was negatively associated with several endurance thresholds and improved regression models beyond V̇O_2_max and sex, with larger incremental contributions than 15-s work metrics when compared in separate one-marker models. Secondary analyses indicated small additional contributions of 15-s work/FFM beyond *v*La_peak_ for some lactate-derived thresholds. These findings suggest that sprint-derived lactate kinetics provide information relevant to threshold power that is not captured by V̇O_2_max alone, but confirmation in longitudinal and more diverse cohorts is required.

**Data Availability Statement**

The data that support the findings of this study are available on reasonable request from the corresponding author. The data are not publicly available due to privacy or ethical restrictions.

**Acknowledgement**

Further studies of the dataset related to this larger project can be found in (Meixner et al. 2025a; Meixner et al. 2024a; Meixner et al. 2026; Meixner et al. 2024b)

**Funding Information**

Authors state no funding involved.

**Conflict of Interest**

Authors state no conflict of interest.

**References**

Aandahl MH, Noordhof DA, Tjønna AE, Sandbakk Ø (2021) Effect of Carbohydrate Content in a Pre-event Meal on Endurance Performance-Determining Factors: A Randomized Controlled Crossover-Trial. Frontiers in Sports and Active Living Volume 3 - 2021. doi:10.3389/fspor.2021.664270

Abt G, Boreham C, Davison G, Jackson R, Jobson S, Wallace E, Williams M (2025) Sample size estimation revisited. J Sports Sci 43 (21):2511–2516. doi:10.1080/02640414.2025.2499403

Adam J, Ohmichen M, Ohmichen E, Rother J, Muller UM, Hauser T, Schulz H (2015) Reliability of the calculated maximal lactate steady state in amateur cyclists. Biol Sport 32 (2):97–102. doi:10.5604/20831862.1134311

Amaro-Gahete FJ, Sanchez-Delgado G, Ruiz JR (2018) Commentary: Contextualising Maximal Fat Oxidation During Exercise: Determinants and Normative Values. Frontiers in Physiology Volume 9 - 2018. doi:10.3389/fphys.2018.01460

Bassett DR, Jr., Howley ET (2000) Limiting factors for maximum oxygen uptake and determinants of endurance performance. Med Sci Sports Exerc 32 (1):70–84. doi:10.1097/00005768-200001000-00012

Beneke R (2003a) Experiment and computer-aided simulation: complementary tools to understand exercise metabolism. Biochem Soc Trans 31 (Pt 6):1263–1266. doi:10.1042/bst0311263

Beneke R (2003b) Maximal lactate steady state concentration (MLSS): experimental and modelling approaches. Eur J Appl Physiol 88 (4-5):361–369. doi:10.1007/s00421-002-0713-2

Benítez-Muñoz JA, Benito PJ, Guisado-Cuadrado I, Cupeiro R, Peinado AB (2024) Differences in the ventilatory thresholds in treadmill according to training status in 971 males and 301 females: a cross-sectional study. European Journal of Applied Physiology. doi:10.1007/s00421-024-05622-z

Benitez-Muñoz JA, Guisado-Cuadrado I, Rojo-Tirado MA, Alcocer-Ayuga M, Romero-Parra N, Peinado AB, Cupeiro R (2024) Females have better metabolic flexibility in different metabolically challenging stimuli. Appl Physiol Nutr Metab. doi:10.1139/apnm-2024-0217

Benítez-Muñoz JA, Rojo-Tirado MÁ, Benito Peinado PJ, Murias JM, González-Lamuño D, Cupeiro R (2025) Greater Relative First and Second Lactate Thresholds in Females Compared With Males: Consideration for Exercise Prescription. International Journal of Sports Physiology and Performance 20 (1):30–36. doi:10.1123/ijspp.2024-0079

Bleicher A, Mader A, Mester J (1998) Zur Interpretation von Laktatleistungskurven–experimentelle Ergebnisse mit computergestützten Nachberechnungen. Spectrum der Sportwissenschaften 10 (1):92–104

Boisseau N, Isacco L (2022) Substrate metabolism during exercise: Sexual dimorphism and women's specificities. Eur J Sport Sci 22 (5):672–683. doi:10.1080/17461391.2021.1943713

Bonen A (2001) The expression of lactate transporters (MCT1 and MCT4) in heart and muscle. European Journal of Applied Physiology 86 (1):6–11. doi:10.1007/s004210100516

Boscá L, Corredor C (1984) Is phosphofructokinase the rate-limiting step of glycolysis? Trends in Biochemical Sciences 9 (9):372–373. doi:https://doi.org/10.1016/0968-0004(84)90214-7

Bouza C (2013) Handbook of Computational Statistics. Concepts and Methods. Investigación Operacional 34 (1):90–91

Brooks GA (2009) Cell-cell and intracellular lactate shuttles. J Physiol 587 (Pt 23):5591–5600. doi:10.1113/jphysiol.2009.178350

Brooks GA (2010) What does glycolysis make and why is it important? Journal of Applied Physiology 108 (6):1450–1451. doi:10.1152/japplphysiol.00308.2010

Brooks GA (2018) The Science and Translation of Lactate Shuttle Theory. Cell Metabolism 27 (4):757–785. doi:10.1016/j.cmet.2018.03.008

Brooks GA, Arevalo JA, Osmond AD, Leija RG, Curl CC, Tovar AP (2022a) Lactate in contemporary biology: a phoenix risen. J Physiol 600 (5):1229–1251. doi:10.1113/jp280955

Brooks GA, Osmond AD, Leija RG, Curl CC, Arevalo JA, Duong JJ, Horning MA (2022b) The blood lactate/pyruvate equilibrium affair. American Journal of Physiology-Endocrinology and Metabolism 322 (1):E34–E43. doi:10.1152/ajpendo.00270.2021

Bundle MW, Weyand PG (2012) Sprint exercise performance: does metabolic power matter? Exerc Sport Sci Rev 40 (3):174–182. doi:10.1097/JES.0b013e318258e1c1

Burtscher J, Millet GP, Burtscher M (2023) Celebrating 100 years of VO2max. Qjm 116 (9):809. doi:10.1093/qjmed/hcad082

Chrzanowski-Smith OJ, Edinburgh RM, Thomas MP, Haralabidis N, Williams S, Betts JA, Gonzalez JT (2020) The day-to-day reliability of peak fat oxidation and FATMAX. European Journal of Applied Physiology 120 (8):1745–1759. doi:10.1007/s00421-020-04397-3

Clark B, Macdermid P (2025) VLa max Correlates Strongly With Glycolytic Performance. Research Quarterly for Exercise and Sport 96:1–8. doi:10.1080/02701367.2025.2481176

Coyle E, Feltner M, Kautz S, Hamilton M, Montain S, Baylor A, Abraham L, Petrek G (1991) Physiological and biomechanical factors associated with elite endurance cycling performance. Medicine & Science in Sports & Exercise 23 (1):93–107

Coyle EF, Coggan AR, Hopper M, Walters TJ (1988) Determinants of endurance in well-trained cyclists. Journal of applied physiology 64 (6):2622–2630

Dahmen T, Byshko R, Saupe D, Röder M, Mantler S (2011) Validation of a model and a simulator for road cycling on real tracks. Sports Engineering 14 (2):95–110

Devries MC (2016) Sex-based differences in endurance exercise muscle metabolism: impact on exercise and nutritional strategies to optimize health and performance in women. Exp Physiol 101 (2):243–249. doi:10.1113/ep085369

di Prampero PE, Ferretti G (1999) The energetics of anaerobic muscle metabolism: a reappraisal of older and recent concepts. Respiration Physiology 118 (2):103–115. doi:https://doi.org/10.1016/S0034-5687(99)00083-3

Dubouchaud H, Butterfield GE, Wolfel EE, Bergman BC, Brooks GA (2000) Endurance training, expression, and physiology of LDH, MCT1, and MCT4 in human skeletal muscle. Am J Physiol Endocrinol Metab 278 (4):E571–579. doi:10.1152/ajpendo.2000.278.4.E571

Dunst AK, Hesse C, Feldmann A, Holmberg HC (2023) A Novel Approach to Determining the Alactic Time Span in Connection with Assessment of the Maximal Rate of Lactate Accumulation in Elite Track Cyclists. Int J Sports Physiol Perform 18 (2):157–163. doi:10.1123/ijspp.2021-0464

Dunst AK, Hesse C, Ueberschär O (2025) Enhancing endurance performance predictions: the role of movement velocity in metabolic simulations demonstrated by cycling cadence. European Journal of Applied Physiology. doi:10.1007/s00421-024-05663-4

Esbjörnsson-Liljedahl M, Bodin K, Jansson E (2002) Smaller muscle ATP reduction in women than in men by repeated bouts of sprint exercise. Journal of Applied Physiology 93 (3):1075–1083. doi:10.1152/japplphysiol.00732.1999

Esteves JV, Stanford KI (2024) Exercise as a tool to mitigate metabolic disease. Am J Physiol Cell Physiol 327 (3):C587–c598. doi:10.1152/ajpcell.00144.2024

Farrell PA, Wilmore JH, Coyle EF, Billing JE, Costill DL (1979) Plasma lactate accumulation and distance running performance. Med Sci Sports 11 (4):338–344

Faude O, Kindermann W, Meyer T (2009) Lactate threshold concepts: how valid are they? Sports Med 39 (6):469–490. doi:10.2165/00007256-200939060-00003

Ferguson BS, Rogatzki MJ, Goodwin ML, Kane DA, Rightmire Z, Gladden LB (2018) Lactate metabolism: historical context, prior misinterpretations, and current understanding. Eur J Appl Physiol 118 (4):691–728. doi:10.1007/s00421-017-3795-6

Ferretti G (2015) Energetics of muscular exercise. Springer,

Ferretti G (2023) Exercise, Respiratory and Environmental Physiology: A Tribute from the School of Milano. Springer Nature,

Ferretti G, di Prampero PE (2026) A reassessment of the energetic significance of blood lactate accumulation during exercise. European Journal of Applied Physiology. doi:10.1007/s00421-026-06134-8

Fischer J, Hävecker F, Ji S, Wahl P, Keller S (2025) Modeling lactate threshold in cycling—influence of sex, maximal oxygen uptake, and cost of cycling in young athletes. European Journal of Applied Physiology. doi:10.1007/s00421-025-05744-y

Glancy B, Kane DA, Kavazis AN, Goodwin ML, Willis WT, Gladden LB (2021) Mitochondrial lactate metabolism: history and implications for exercise and disease. J Physiol 599 (3):863–888. doi:10.1113/jp278930

Haase R, Dunst AK, Nitzsche N (2024) The Influence of Pedaling Frequency on Blood Lactate Accumulation in Cycling Sprints. Int J Sports Med. doi:10.1055/a-2255-5254

Hagberg JM, Coyle EF (1983) Physiological determinants of endurance performance as studied in competitive racewalkers. Medicine and Science in Sports and Exercise 15 (4):287–289

Hargreaves M, Spriet LL (2020) Skeletal muscle energy metabolism during exercise. Nature Metabolism 2 (9):817–828. doi:10.1038/s42255-020-0251-4

Harriss DJ, Atkinson G (2009) International Journal of Sports Medicine - ethical standards in sport and exercise science research. Int J Sports Med 30 (10):701–702. doi:10.1055/s-0029-1237378

Hartmann U, Mader A, Hollmann W (1990) Heart rate and lactate during endurance training programs in rowing and its relation to the duration of exercise by top elite rowers. FISA Coach 1 (1):1–4

Hauser T, Adam J, Schulz H (2014) Comparison of calculated and experimental power in maximal lactate-steady state during cycling. Theor Biol Med Model 11:25. doi:10.1186/1742-4682-11-25

Heck H, Schulz H, Bartmus U (2003) Diagnostics of anaerobic power and capacity. European Journal of Sport Science 3 (3):1–23. doi:10.1080/17461390300073302

Heck H, Wackerhage H (2024) The origin of the maximal lactate steady state (MLSS). BMC Sports Science, Medicine and Rehabilitation 16 (1):36. doi:10.1186/s13102-024-00827-3

Held S, Rappelt L, Brockherde J, Donath L (2023) Reliability of the Maximal Lactate Accumulation Rate in Rowers. Int J Sports Med. doi:10.1055/a-2206-4959

Iannetta D, Inglis EC, Fullerton C, Passfield L, Murias JM (2018) Metabolic and performance-related consequences of exercising at and slightly above MLSS. Scandinavian Journal of Medicine & Science in Sports 28 (12):2481–2493. doi:https://doi.org/10.1111/sms.13280

Jamnick NA, Pettitt RW, Granata C, Pyne DB, Bishop DJ (2020) An Examination and Critique of Current Methods to Determine Exercise Intensity. Sports Med 50 (10):1729–1756. doi:10.1007/s40279-020-01322-8

Jeacocke NA, Burke LM (2010) Methods to Standardize Dietary Intake Before Performance Testing. International Journal of Sport Nutrition and Exercise Metabolism 20 (2):87–103. doi:10.1123/ijsnem.20.2.87

Jeukendrup A, Achten J (2001) Fatmax : A new concept to optimize fat oxidation during exercise? European Journal of Sport Science 1 (5):1–5. doi:10.1080/17461390100071507

Jeukendrup AE, Wallis GA (2005) Measurement of Substrate Oxidation During Exercise by Means of Gas Exchange Measurements. Int J Sports Med 26 (S 1):S28–S37. doi:10.1055/s-2004-830512

Ji S, Sommer A, Bloch W, Wahl P (2021) Comparison and Performance Validation of Calculated and Established Anaerobic Lactate Thresholds in Running. Medicina (Kaunas) 57 (10). doi:10.3390/medicina57101117

Johansen JM, Støa E, Sunde A, Rønnestad B, Helgerud J, Støren O (2025) Elite Aerobic Endurance Performance: Is It Really Related to Lactate Threshold Expressed Relative to Peak Oxygen Uptake? International Journal of Sports Physiology and Performance 20:1–5. doi:10.1123/ijspp.2024-0528

Joyner MJ (1991) Modeling: optimal marathon performance on the basis of physiological factors. J Appl Physiol (1985) 70 (2):683–687. doi:10.1152/jappl.1991.70.2.683

Keir DA, Iannetta D, Mattioni Maturana F, Kowalchuk JM, Murias JM (2022) Identification of Non-Invasive Exercise Thresholds: Methods, Strategies, and an Online App. Sports Medicine 52 (2):237–255. doi:10.1007/s40279-021-01581-z

Kitaoka Y, Hoshino D, Hatta H (2012) Monocarboxylate transporter and lactate metabolism. The Journal of Physical Fitness and Sports Medicine 1 (2):247–252. doi:10.7600/jpfsm.1.247

Lakens D (2022) Sample Size Justification. Collabra: Psychology 8 (1). doi:10.1525/collabra.33267

Lanaspeze G, Best M, Guilbert B, Cavoret J, Duval A, Manin L, Ville F (2025) Identification of key parameter influence on efficiency of chain drive for track cycling− Preliminary results. Mechanics & Industry 26:21

Langley J, Haase R, Nitzsche N, Porter M (2025) Methodological approaches in testing maximal lactate accumulation rate - νLamax: A systematic review. Journal Of Science & Cycling 14:1–40. doi:10.28985/1425.jsc.09

Langley JO, Ng SC, Todd EE, Porter MS (2024) V˙ La(max): determining the optimal test duration for maximal lactate formation rate during all-out sprint cycle ergometry. Eur J Appl Physiol. doi:10.1007/s00421-024-05456-9

Leija RG, Arevalo JA, Xing D, Vázquez-Medina JP, Brooks GA (2025) The mitochondrial lactate oxidation complex: endpoint for carbohydrate carbon disposal. American Journal of Physiology-Endocrinology and Metabolism 328 (1):E126–E136. doi:10.1152/ajpendo.00306.2024

Lucía A, Pardo J, Durántez A, Hoyos J, Chicharro JL (1998) Physiological Differences Between Professional and Elite Road Cyclists. Int J Sports Med 19 (05):342–348. doi:10.1055/s-2007-971928

Mader A (1984) Eine Theorie zur Berechnung der Dynamik und des steady state von Phosphorylierungszustand und Stoffwechselaktivität der Muskelzelle als Folge des Energiebedarfs. na,

Mader A (2003) Glycolysis and oxidative phosphorylation as a function of cytosolic phosphorylation state and power output of the muscle cell. Eur J Appl Physiol 88 (4-5):317–338. doi:10.1007/s00421-002-0676-3

Mader A, Heck H (1986) A Theory of the Metabolic Origin of "Anaerobic Threshold". Int J Sports Med 7:20

Margaria R, Cerretelli P, Mangili F (1964) BALANCE AND KINETICS OF ANAEROBIC ENERGY RELEASE DURING STRENUOUS EXERCISE IN MAN. J Appl Physiol 19:623–628. doi:10.1152/jappl.1964.19.4.623

Marsh AP, MARTIN PE, FOLEY KO (2000) Effect of cadence, cycling experience, and aerobic power on delta efficiency during cycling. Medicine & Science in Sports & Exercise 32 (9):1630–1634

Maunder E, Plews DJ, Kilding AE (2018) Contextualising Maximal Fat Oxidation During Exercise: Determinants and Normative Values. Frontiers in Physiology Volume 9 - 2018. doi:10.3389/fphys.2018.00599

McCrum C, van Beek J, Schumacher C, Janssen S, Van Hooren B (2022) Sample size justifications in Gait & Posture. Gait & Posture 92:333–337. doi:https://doi.org/10.1016/j.gaitpost.2021.12.010

McLester CN, Nickerson BS, Kliszczewicz BM, McLester JR (2020) Reliability and Agreement of Various InBody Body Composition Analyzers as Compared to Dual-Energy X-Ray Absorptiometry in Healthy Men and Women. Journal of Clinical Densitometry 23 (3):443–450. doi:https://doi.org/10.1016/j.jocd.2018.10.008

Meixner B, Filipas L, Holmberg HC, Sperlich B (2025a) Zone 2 Intensity: A Critical Comparison of Individual Variability in Different Submaximal Exercise Intensity Boundaries. Transl Sports Med 2025 (1):2008291. doi:10.1155/tsm2/2008291

Meixner B, Matzka M, Sperlich B (2025b) Comparison of maximal glycolytic rate from ergometer to on-water sprinting in elite canoe polo players. Appl Physiol Nutr Metab 50:1–10. doi:10.1139/apnm-2024-0450

Meixner B, Nusser V, Koehler K, Sablain M, Boone J, Sperlich B (2024a) Reliability of power output, maximal rate of capillary blood lactate accumulation, and phosphagen contribution time following 15-s sprint cycling in amateur cyclists. Physiol Rep 12 (10):e16086. doi:10.14814/phy2.16086

Meixner B, Schaffarczyk M, Sperlich B (2026) Method Modulates: Protocol Choices Shape Sex Differences in the Determination of Exercise intensity. American Journal of Physiology-Regulatory, Integrative and Comparative Physiology 0 (0):null. doi:10.1152/ajpregu.00266.2025

Meixner B, Stegmaier J, Renner P, Koehler K, Yang WH, Sperlich B (2025c) Supplementation of Creatine Monohydrate Improves Sprint Performance but Has no Effect on Glycolytic Contribution: A Nonrandomized, Placebo-Controlled Crossover Trial in Trained Cyclists. Curr Dev Nutr 9 (2):104561. doi:10.1016/j.cdnut.2025.104561

Meixner BJ, Nusser V, Koehler K, Sablain M, Boone J, Sperlich B (2024b) Relationship of peak capillary blood lactate accumulation and body composition in determining the mechanical energy equivalent of lactate during sprint cycling. Eur J Appl Physiol 124 (11):3399–3407. doi:10.1007/s00421-024-05529-9

Midgley A, Carroll S (2009) Emergence of the verification phase procedure for confirming ‘true’VO2max. Scandinavian journal of medicine & science in sports 19 (3):313–322

Millet GP, Burtscher J, Bourdillon N, Manferdelli G, Burtscher M, Sandbakk Ø (2023) The V˙O2max Legacy of Hill and Lupton (1923)-100 Years On. Int J Sports Physiol Perform 18 (11):1362–1365. doi:10.1123/ijspp.2023-0229

Mor I, Cheung EC, Vousden KH (2011) Control of glycolysis through regulation of PFK1: old friends and recent additions. Cold Spring Harb Symp Quant Biol 76:211–216. doi:10.1101/sqb.2011.76.010868

Nitzsche N, Baumgärtel L, Schulz H (2018) Comparison of Maximum Lactate Formation Rates in Ergometer Sprint and Maximum Strength Loads. Deutsche Zeitschrift f¸r Sportmedizin 69 (1):13–18. doi:10.5960/dzsm.2017.312

Nolte S, Rein R, Quittmann OJ (2023) Data Processing Strategies to Determine Maximum Oxygen Uptake: A Systematic Scoping Review and Experimental Comparison with Guidelines for Reporting. Sports Medicine 53 (12):2463–2475. doi:10.1007/s40279-023-01903-3

Oosthuyse T, Bosch AN (2006) Influence of menstrual phase on ventilatory responses to submaximal exercise : original research article. South African Journal of Sports Medicine 18 (2):31–37. doi:doi:10.10520/EJC66960

Pallarés JG, Morán-Navarro R, Ortega JF, Fernández-Elías VE, Mora-Rodriguez R (2016) Validity and Reliability of Ventilatory and Blood Lactate Thresholds in Well-Trained Cyclists. PLOS ONE 11 (9):e0163389. doi:10.1371/journal.pone.0163389

Poffé C, Van Dael K, Van Schuylenbergh R (2024) INSCYD physiological performance software is valid to determine the maximal lactate steady state in male and female cyclists. Frontiers in Sports and Active Living 6. doi:10.3389/fspor.2024.1376876

Poole DC, Rossiter HB, Brooks GA, Gladden LB (2021) The anaerobic threshold: 50+ years of controversy. The Journal of Physiology 599 (3):737–767. doi:https://doi.org/10.1113/JP279963

Purdom T, Kravitz L, Dokladny K, Mermier C (2018) Understanding the factors that effect maximal fat oxidation. J Int Soc Sports Nutr 15:3. doi:10.1186/s12970-018-0207-1

Quittmann OJ (2025) Maximal lactate accumulation rate ($${\dot{\text{c}}}$$Lamax): Current evidence and future directions for exercise testing and training. European Journal of Applied Physiology. doi:10.1007/s00421-025-06022-7

Quittmann OJ, Foitschik T, Vafa R, Freitag FJ, Sparmann N, Nolte S, Abel T (2022) Is Maximal Lactate Accumulation Rate Promising for Improving 5000-m Prediction in Running? Int J Sports Med 44 (04):268–279. doi:10.1055/a-1958-3876

Rotstein A, Dotan R, Zigel L, Greenberg T, Benyamini Y, Falk B (2007) The effect of pre-test carbohydrate ingestion on the anaerobic threshold, as determined by the lactate-minimum test. Applied Physiology, Nutrition, and Metabolism 32:1058–1064. doi:10.1139/H07-066

Sablain M, Sperlich B, Meixner B, Caen K, Vermeire K, Boone J (2025) Evaluating the maximal rate of lactate accumulation and estimated maximal lactate steady state in cycling. Eur J Appl Physiol 125 (8):2173–2183. doi:10.1007/s00421-025-05751-z

San-Millan I, Brooks GA (2018) Assessment of Metabolic Flexibility by Means of Measuring Blood Lactate, Fat, and Carbohydrate Oxidation Responses to Exercise in Professional Endurance Athletes and Less-Fit Individuals. Sports Med 48 (2):467–479. doi:10.1007/s40279-017-0751-x

Seyedi R, Tayebi SM, Zhang D, Yiming Q (2024) The role of monocarboxylate transporter-1 and -4 in exercise and training: A mini-review article. Science & Sports 39 (2):144–152. doi:https://doi.org/10.1016/j.scispo.2022.11.009

Spriet LL, Howlett RA, Heigenhauser GJ (2000) An enzymatic approach to lactate production in human skeletal muscle during exercise. Med Sci Sports Exerc 32 (4):756–763. doi:10.1097/00005768-200004000-00007

Tarnopolsky MA, Ruby BC (2001) Sex differences in carbohydrate metabolism. Curr Opin Clin Nutr Metab Care 4 (6):521–526. doi:10.1097/00075197-200111000-00010

Thomas C, Perrey S, Lambert K, Hugon G, Mornet D, Mercier J (2005) Monocarboxylate transporters, blood lactate removal after supramaximal exercise, and fatigue indexes in humans. J Appl Physiol (1985) 98 (3):804–809. doi:10.1152/japplphysiol.01057.2004

Thomas DT, Erdman KA, Burke LM (2016) Position of the Academy of Nutrition and Dietetics, Dietitians of Canada, and the American College of Sports Medicine: Nutrition and Athletic Performance. Journal of the Academy of Nutrition and Dietetics 116 (3):501–528. doi:https://doi.org/10.1016/j.jand.2015.12.006

Thron M, Woll A, Doller L, Quittmann OJ, Härtel S, Ruf L, Altmann S (2024) Physiological and Locomotor Profiling Enables to Differentiate Between Sprinters, 400-m Runners, and Middle-Distance Runners. J Strength Cond Res 38 (8):1419–1427. doi:10.1519/jsc.0000000000004801

Tripp TR, Kontro H, Gillen JB, MacInnis MJ (2025) Fit for comparison: controlling for cardiorespiratory fitness in exercise physiology studies of sex as a biological variable. The Journal of Physiology 603 (8):2219–2230. doi:https://doi.org/10.1113/JP287735

Vigh-Larsen JF, Ørtenblad N, Spriet LL, Overgaard K, Mohr M (2021) Muscle Glycogen Metabolism and High-Intensity Exercise Performance: A Narrative Review. Sports Medicine 51 (9):1855–1874. doi:10.1007/s40279-021-01475-0

Wackerhage H, Gehlert S, Schulz H, Weber S, Ring-Dimitriou S, Heine O (2022) Lactate Thresholds and the Simulation of Human Energy Metabolism: Contributions by the Cologne Sports Medicine Group in the 1970s and 1980s. Front Physiol 13:899670. doi:10.3389/fphys.2022.899670

Wackerhage H, Kabasakalis A, Seiler S, Heck H (2025) Is the vLamax for Glycolysis What the VO2max is for Oxidative Phosphorylation? Sports Medicine. doi:10.1007/s40279-025-02259-6

Wahl P, Ji S (2026) From diagnostics to prediction: development and validation of a multi-domain power-duration model. European Journal of Applied Physiology. doi:10.1007/s00421-026-06142-8

Yoshida T, Chida M, Ichioka M, Suda Y (1987) Blood lactate parameters related to aerobic capacity and endurance performance. European journal of applied physiology and occupational physiology 56 (1):7–11

Zuo J, Tang J, Lu M, Zhou Z, Li Y, Tian H, Liu E, Gao B, Liu T, Shao P (2021) Glycolysis Rate-Limiting Enzymes: Novel Potential Regulators of Rheumatoid Arthritis Pathogenesis. Front Immunol 12:779787. doi:10.3389/fimmu.2021.779787

Figure 1: Time course of study design.

Figure 2: Pearson and partial Pearson correlations between thresholds and sprint test parameters. Abbreviations: FFM = fat-free mass, *v*La_peak_ = maximal lactate accumulation rate, PFO = peak fat oxidation, Fat_max_ = power output associated with maximal fat oxidation, P_LaWmin_ = minimal lactate equivalent, P_2_ = fixed 2 mmol·L^-1^lactate threshold, P_+0.5_ = individual lactate threshold at minimum value + 0.5 mmol·L^-1^, P_4_ = fixed 4 mmol·L^-1^lactate threshold, P_+1.5_ = individual lactate threshold at minimum value + 1.5 mmol·L^-1^, P_VT1_ = power output at ventilatory threshold 1, P_VT2_ = power output at ventilatory threshold 2. *indicates significant correlations

Figure 3: Visual representation of influence of *v*La_peak_ on A) P_VT1_ , P_VT2_ , by including V̇O_2_max, *v*La_peak_ as predictors.

|  | **Total (n=50)** | **Male (n=30)** | **Female (n=20)** |  |
| --- | --- | --- | --- | --- |
| **Age [y]** | 31.2±7.8 | 34.3±7.8 | 26.4±5.0 |  |
| **Height [cm]** | 177±9.3 | 183±7.0 | 170±6.6 |  |
| **Body Mass [kg]** | 71.7±12.4 | 78.6±10.3 | 61.4±6.6 |  |
| **Fat-free Mass [kg]** | 61.9±11.5 | 69.2±7.9 | 50.9±5.9 |  |

Table 1: Anthropometric data for total study population and female and male group

|  | **Total (n=50)** | **Male (n=30)** | **Female (n=20)** |
| --- | --- | --- | --- |
| **15s-work [kJ]** | 11.373±2.927 | 13.131±2.325 | 8.736±1.285 |
| **15s-work/FFM [J/kg]** | 182±22 | 190±24.7 | 172±10.6 |
| **ΔLa [mmol·L^-1^]** | 6.28±1.57 | 6.69±1.68 | 5.66±1.18 |
| ***v*La_peak_ [mmol·L^-1^·s^-1^]** | 0.42±0.11 | 0.45±0.11 | 0.38±0.08 |
| **PFO [g/min]** | 0.38±0.15 | 0.420±152 | 0.327±118 |
| **PFO [mg/min/kg FFM]** | 5.37±1.96 | 5.38±2.01 | 5.36±1.94 |
| **Fat_max_ [W]** | 150±25 | 150±62.5 | 125±0 |
| **P_LaWmin_ [W]** | 163±40 | 186±30 | 128±25 |
| **P_+0.5_ [W]** | 196±48 | 225±36 | 153±28 |
| **P_2_ [W]** | 208±54 | 239±43 | 162±30 |
| **P_+1.5_ [W]** | 224±52 | 256±38 | 177±28 |
| **P_4_ [W]** | 248±57 | 283±43 | 196±30 |
| **P_VT1_ [W]** | 200±46 | 224±40 | 163±24 |
| **P_VT2_ [W]** | 250±50 | 279±40 | 206±24 |

Table 2: Mean±SD for all relevant parameters determined in the sprint and incremental step test, Median ± MAD for Fat_max_. Abbreviations: FFM = fat-free mass, ΔLa = maximal lactate accumulation, *v*La_peak_ = maximal lactate accumulation rate, PFO = peak fat oxidation, Fat_max_ = power output associated with maximal fat oxidation, P_LaWmin_ = minimal lactate equivalent, P_2_ = fixed 2 mmol·L^-1^lactate threshold, P_+0.5_ = individual lactate threshold at minimum value + 0.5 mmol·L^-1^, P_4_ = fixed 4 mmol·L^-1^ lactate threshold, P_+1.5_ = individual lactate threshold at minimum value + 1.5 mmol·L^-1^, P_VT1_ = power output at ventilatory threshold 1, P_VT2_ = power output at ventilatory threshold 2.

| **Threshold** | **Parameter added to M_0_** | **B** | **β** | **p** | **R^2^ (full)** | **ΔR^2^ (vs M_o_)** | **p(ΔR^2^)** |
| --- | --- | --- | --- | --- | --- | --- | --- |
| Fat_max_ | *v*La_peak_ | -189.724 | -0.478 | <0.001 | 0.629 | 0.203 | <0.001 |
| Fat_max_ | 15s-work | -0.008 | -0.551 | 0.013 | 0.540 | 0.114 | 0.001 |
| Fat_max_ | 15s-work/FFM | -0.773 | -0.409 | 0.006 | 0.565 | 0.139 | <0.001 |
| P_LaWmin_ | *v*La_peak_ | -99.643 | -0.263 | 0.002 | 0.776 | 0.062 | <0.001 |
| P_LaWmin_ | 15s-work | -0.004 | -0.281 | 0.026 | 0.744 | 0.030 | 0.025 |
| P_LaWmin_ | 15s-work/FFM | -0.178 | -0.099 | 0.155 | 0.723 | 0.008 | 0.252 |
| P_+0.5_ | *v*La_peak_ | -127.403 | -0.278 | <0.001 | 0.850 | 0.069 | <0.001 |
| P_+0.5_ | 15s-work | -0.005 | -0.292 | 0.018 | 0.814 | 0.032 | 0.007 |
| P_+0.5_ | 15s-work/FFM | -0.301 | -0.138 | 0.036 | 0.798 | 0.016 | 0.065 |
| P_2_ | *v*La_peak_ | -181.466 | -0.352 | <0.001 | 0.857 | 0.110 | <0.001 |
| P_2_ | 15s-work | -0.006 | -0.327 | 0.012 | 0.787 | 0.040 | 0.005 |
| P_2_ | 15s-work/FFM | -0.568 | -0.231 | 0.002 | 0.791 | 0.044 | 0.003 |
| P_+1.5_ | *v*La_peak_ | -137.248 | -0.279 | <0.001 | 0.879 | 0.069 | <0.001 |
| P_+1.5_ | 15s-work | -0.004 | -0.243 | 0.035 | 0.832 | 0.022 | 0.018 |
| P_+1.5_ | 15s-work/FFM | -0.311 | -0.133 | 0.037 | 0.825 | 0.015 | 0.056 |
| P_4_ | *v*La_peak_ | -167.516 | -0.306 | <0.001 | 0.891 | 0.083 | <0.001 |
| P_4_ | 15s-work | -0.005 | -0.232 | 0.046 | 0.828 | 0.020 | 0.025 |
| P_4_ | 15s-work/FFM | -0.438 | -0.168 | 0.011 | 0.831 | 0.023 | 0.015 |
| P_VT1_ | *v*La_peak_ | -132.906 | -0.306 | <0.001 | 0.798 | 0.083 | <0.001 |
| P_VT1_ | 15s-work | -0.004 | -0.239 | 0.059 | 0.736 | 0.021 | 0.059 |
| P_VT1_ | 15s-work/FFM | -0.485 | -0.234 | 0.005 | 0.761 | 0.046 | 0.005 |
| P_VT2_ | *v*La_peak_ | -125.579 | -0.262 | <0.001 | 0.880 | 0.061 | <0.001 |
| P_VT2_ | 15s-work | -0.004 | -0.209 | 0.037 | 0.836 | 0.017 | 0.037 |
| P_VT2_ | 15s-work/FFM | -0.465 | -0.203 | 0.002 | 0.854 | 0.034 | 0.002 |

Table 3: Results for model comparison of M_0_ with V̇O_2_max and sex as predictors and adding either *v*La_peak_, 15s-work or 15s-work/FFM as predictors. FFM= fat-free mass, *v*La_peak_ = maximal lactate accumulation rate, PFO = peak fat oxidation, Fat_max_ = power output associated with maximal fat oxidation, P_LaWmin_ = minimal lactate equivalent, P_2_ = fixed 2 mmol·L^-1^lactate threshold, P_+0.5_ = individual lactate threshold at minimum value + 0.5 mmol·L^-1^, P_4_ = fixed 4 mmol·L^-1^ lactate threshold, P_+1.5_ = individual lactate threshold at minimum value + 1.5 mmol·L^-1^, P_VT1_ = power output at ventilatory threshold 1, P_VT2_ = power output at ventilatory threshold 2.

| **Threshold** | | **Parameter added to M_1_** | | **B** | | **β** | | **p** | | **R^2^ (full)** | **ΔR^2^ (vs M_1_)** | **p(ΔR^2^)** |  |
| --- | --- | --- | --- | --- | --- | --- | --- | --- | --- | --- | --- | --- | --- |
| Fat_max_ | | 15s-work | | -0.128 | | -0.551 | | 0.706 | | 0.630 | 0.002 | 0.667 |  |
| Fat_max_ | | 15s-work/FFM | | -0.002 | | -0.409 | | 0.466 | | 0.635 | 0.006 | 0.399 |  |
| P_LaWmin_ | | 15s-work | | 0.497 | | -0.281 | | 0.036 | | 0.801 | 0.025 | 0.021 |  |
| P_LaWmin_ | | 15s-work/FFM | | -0.001 | | -0.099 | | 0.772 | | 0.777 | 0.001 | 0.721 | |
| P_+0.5_ | | 15s-work | | 0.455 | | -0.292 | | 0.043 | | 0.865 | 0.015 | 0.033 |  |
| P_+0.5_ | | 15s-work/FFM | | -0.001 | | -0.138 | | 0.772 | | 0.851 | 0.001 | 0.697 | |
| P_2_ | | 15s-work | | 0.303 | | -0.327 | | 0.290 | | 0.862 | 0.005 | 0.203 |  |
| P_2_ | | 15s-work/FFM | | 0.000 | | -0.231 | | 0.951 | | 0.857 | 0.000 | 0.932 |  |
| P_+1.5_ | | 15s-work | | 0.522 | | -0.243 | | 0.023 | | 0.896 | 0.017 | 0.010 |  |
| P_+1.5_ | 15s-work/FFM | 0.001 | -0.133 | | 0.815 | | 0.879 | | 0.000 | | 0.749 | |  |
| P_4_ | | 15s-work | | 0.494 | | -0.232 | | 0.068 | | 0.903 | 0.012 | 0.023 |  |
| P_4_ | | 15s-work/FFM | | 0.002 | | -0.168 | | 0.521 | | 0.893 | 0.002 | 0.362 |  |
| P_VT1_ | | 15s-work | | 0.001 | | 0.082 | | 0.557 | | 0.800 | 0.002 | 0.557 |  |
| P_VT1_ | | 15s-work/FFM | | 0.051 | | 0.025 | | 0.830 | | 0.798 | 0.000 | 0.830 |  |
| P_VT2_ | | 15s-work | | 0.001 | | 0.062 | | 0.563 | | 0.881 | 0.001 | 0.563 | |
| P_VT2_ | | 15s-work/FFM | | 0.032 | | 0.014 | | 0.874 | | 0.881 | 0.000 | 0.874 |  |

Table 4: Results for nested model comparison of M_1_ with V̇O_2_max, *v*La_peak_ and sex as predictors and adding either 15s-work or 15s-work/FFM as predictors. FFM= fat-free mass, *v*La_peak_ = maximal lactate accumulation rate, PFO = peak fat oxidation, Fat_max_ = power output associated with maximal fat oxidation, P_LaWmin_ = minimal lactate equivalent, P_2_ = fixed 2 mmol·L^-1^lactate threshold, P_+0.5_ = individual lactate threshold at minimum value + 0.5 mmol·L^-1^, P_4_ = fixed 4 mmol·L^-1^ lactate threshold, P_+1.5_ = individual lactate threshold at minimum value + 1.5 mmol·L^-1^, P_VT1_ = power output at ventilatory threshold 1, P_VT2_ = power output at ventilatory threshold 2.
